# State-of-the-art of situation recognition systems for intraoperative procedures

**DOI:** 10.1007/s11517-022-02520-4

**Published:** 2022-02-17

**Authors:** D. Junger, S. M. Frommer, O. Burgert

**Affiliations:** School of Informatics, Research Group Computer Assisted Medicine (CaMed), Reutlingen University, Alteburgstr. 150, 72762 Reutlingen, Germany

**Keywords:** Situation recognition, Situation awareness, Operating room, Applicability, Transferability

## Abstract

One of the key challenges for automatic assistance is the support of actors in the operating room depending on the status of the procedure. Therefore, context information collected in the operating room is used to gain knowledge about the current situation. In literature, solutions already exist for specific use cases, but it is doubtful to what extent these approaches can be transferred to other conditions. We conducted a comprehensive literature research on existing situation recognition systems for the intraoperative area, covering 274 articles and 95 cross-references published between 2010 and 2019. We contrasted and compared 58 identified approaches based on defined aspects such as used sensor data or application area. In addition, we discussed applicability and transferability. Most of the papers focus on video data for recognizing situations within laparoscopic and cataract surgeries. Not all of the approaches can be used online for real-time recognition. Using different methods, good results with recognition accuracies above 90% could be achieved. Overall, transferability is less addressed. The applicability of approaches to other circumstances seems to be possible to a limited extent. Future research should place a stronger focus on adaptability. The literature review shows differences within existing approaches for situation recognition and outlines research trends. Applicability and transferability to other conditions are less addressed in current work.

**Graphical abstract:**

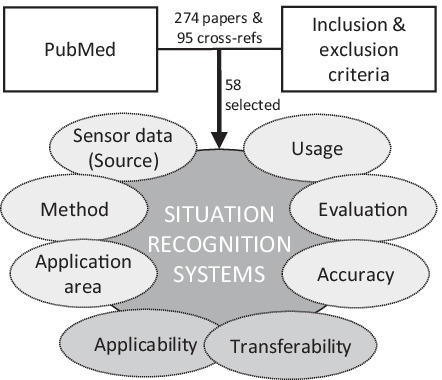

**Supplementary Information:**

The online version contains supplementary material available at 10.1007/s11517-022-02520-4.

## Introduction

The vision of intraoperative context-aware systems is the automatic support of processes in the operating room (OR) based on the course of the operation and the current process step. Context-aware systems use context information of the environment to provide services relevant to the current task [51]. This would allow surgeons and their team to be supported in a highly targeted manner. The application area of such systems is comprehensive and ranges from the situation-dependent provision of context-relevant information, like preoperative images (e.g., filter information automatically [26]), to the automatic execution of defined subtasks, such as informing about delays (e.g., estimating the intervention time [17]), or semi-automatic generation of OR reports. To realize these kinds of systems, it is crucial to detect the actual situation reliably to provide information suitable to the situation. For this recognition task, a system that is capable of deriving a “situation” information from sensor input is needed. We define this system as a “situation recognition system” to provide situation and context awareness.

Three of the main aspects of context awareness for surgical support are formal modeling of processes, intraoperative tracking and processing, and implementation of context-aware equipment [18]. The basis for context-aware systems in the OR is knowledge of the intraoperative procedures and the current situation of the operation (situation awareness). For this, the current situation must be recognized at specific granularities [37]. Lalys and Jannin [33] differentiate between procedures, phases, steps, activities, and motions in ascending granularity. Similarly, the ontology of Nakawala et al. [47] includes the granularities: phases, steps, and actions (in our case defined as activities). Activities can be represented by different information [38, 45]. Lalys et al. [38] describe an activity as a triplet of action, surgical tool, and anatomical structure, whereas Meißner et al. [45] define a 5-tuple: actor, used body part, used surgical instrument, surgical action, and treated structure. To identify the surgical workflow, data from available sensors in the OR are used [13] to detect different information about the context (instruments, persons, anatomical structures, etc.). Sensors can be of any kind like location sensors, endoscopy images, and medical device communication messages. The acquired data is then used in interpretation systems, in our case defined as situation recognition systems, to automatically recognize the situation in the OR. A situation can be represented internally in various forms (e.g., ontologies, state machines). For situation interpretation, approaches like machine learning techniques or formal methods can be applied [26]. The information about the current situation can then be used for various use cases, such as situation-related displays or workflow automation. The recognition of situations is the foundation for supportive context-aware systems and therefore of significant relevance in research to enable context-aware systems.

For different interventions, solutions already exist in the research literature which enable the recognition of the current situation. But it is doubtful to what extent these approaches can react to process and technology changes to be transferred to other circumstances, like more complex interventions or sensor systems. This would be advantageous considering the additional effort for commissioning, maintenance, etc. for different systems. To examine which approaches already exist to what extent, an overview and analysis of the current state of research could highlight similarities and differences between existing systems and examine aspects of applicability and transferability.

In the area of situation recognition, reviews of literature that deal with data acquisition techniques or situation interpretation already exist. The work of Kranzfelder et al. [32] presents a literature review of 2010 with currently promising technologies for real-time data acquisition in the OR. The feasibility results indicate that methods for continuous sensor-based data acquisition and online analysis based on device information are suitable. Instrument recognition via barcodes, tracking of persons via RFID, and emotion recognition via speech seem promising. Similar work by Pernek et al. [51] deals with a literature review of 2015 with currently existing approaches for automatic context recognition during surgical interventions. The comparison results indicate that there is a large discrepancy in methods, depending on the type of surgical context that is recognized. For future approaches, more elaborate evaluations should be carried out under real conditions. The work of Padoy [49] includes a literature review of 2019 with current machine and deep learning techniques for contextual analysis during interventions, using video data from endoscopes or ceiling-mounted cameras. The results show to what extent videos can be used to recognize phases, instruments, and persons. One of the challenges for current developments is to scale methods to more intervention types and more granular activities.

Other work in the field of situation awareness focuses on specific recognition strategies in defined application areas or summarizes their results. The work of Bouget et al. [7] presents a comprehensive overview of recognition possibilities of vision-based and markerless instruments. The focus is the analysis of available data sets, the comparison of recognition methods, and the analysis of validation techniques. The overview aims to identify key challenges and fields for further development. The work of Ahmidi et al. [1] includes a comparison of different strategies for gesture recognition to evaluate surgical skills. Also, a data set and a consistent methodology for performance assessment are described. Based on the data set, an evaluation of surgical techniques for gesture recognition is performed to provide an overview and recommendations for research. The work of Stauder et al. [56] presents their methods for workflow recognition intending to use this knowledge for context-aware systems. Subsequently, possible future applications, such as context-aware visualizations, as well as a context-aware OR of the future are discussed.

The research works mentioned above provide an overview of techniques, methods, etc. in the context of situation awareness, focusing on different aspects and discussion goals. The review papers do not show any current evaluation of full, closed systems and their applicability. The goal of this research is an up-to-date overview of approaches for automatic information acquisition and analysis in the OR. Therefore, current situation recognition techniques to recognize the current situation of an intervention (cutting-suture) will be presented in a clear and categorized manner. For this purpose, research work is used which presents closed systems for the recognition of the surgical situation on different granularities. The systems will be compared and discussed with regard to relevant aspects such as used sensor technology, area of application, or real-time capability. Focus is also the applicability and transferability of the solutions to different surgical and sensory contexts to examine if the solutions are adaptable to other circumstances. The result provides an assessment of the current state of research in the field of situation awareness in the OR. To the best of our knowledge, no other research group has presented a review of closed situation recognition systems for intraoperative procedures of the last ten years clearly and concisely and in relation to each other, with a focus on applicability and transferability.

## Methods

A literature search of scientific publications on situation recognition technologies in the operative field was carried out, essentially following the PRISMA 2009 guidelines. We adapted the procedure from the method described by Pernek et al. [51]. The PubMed research database was queried extensively for search terms such as “situation recognition”, “context recognition”, “workflow recognition”, “phase recognition”, “step recognition”, and “activity recognition” in combination with “operating room”, “operating theatre”, and “surgery” in title/abstract (query: “{situation, context} aware(-ness)”, “{situation, context, workflow, process, phase, step, activity, action, instrument, anatomy} {recognition, detection, identification}” AND “operating {room, theatre}”, “surgery”, “surgical”). The focus was on the identification of papers that address recognition strategies and technologies in the context of situations, and the papers are filtered according to their application in the intraoperative area. Only recent papers published within the last 10 years (2010–2019), written in English, and available in full text have been taken into account. Papers with electronic pre-print and print publication in 2020 were included, as far as they could be covered by the search. The last search was conducted on 20.01.2020, where we found 274 papers.

As the next step, we analyzed the titles and abstracts of the identified papers with regard to the topic of situation awareness in the OR to identify those that appear relevant. The focus was set on recognition strategies and technologies for achieving situation awareness in the intraoperative area, regardless of whether instruments, phases, or other aspects are recognized, to get a first overview of any recognition approaches. The 54 identified papers were divided into categories: relevant, adjacent (may be relevant), and review. To include publications not covered by the search terms, we analyzed the references of the papers categorized as relevant and included corresponding papers (95 references) with appropriate title and abstract in the overall list of papers. Papers of the cross-referencing were not re-examined for further references.

After filtering and cross-referencing, we examined the paper’s contents to create a final selection of identified papers. The selection was made based on appropriate topics and sufficient information. Because the focus of this work was set on full, closed situation recognition systems (i.e., recognition of phases, steps, and activities), regardless of the support for which they are ultimately used, we excluded all papers that fall below the granularity of activities (i.e., gestures) or recognize instruments or persons and do not have a recognized phase or similar as result. Papers with a different focus (e.g., not automatic) and missing information (e.g., accuracy) were not considered. A total of 58 papers were finally identified for the literature review. To the best of our knowledge, these are complete according to our research procedure.

To present the state-of-the-art clearly and concisely, a table was created. Therefore, we grouped the papers by the granularity of the used recognition, as this shows which possibilities currently exist for situation awareness in different levels of detail. For the tabular representation, we defined aspects based on [32] and [51], as shown in Table 1. Due to the different levels of detail and the partly ambiguous or incomplete information, we summarized the contents of the papers according to the following procedure.

**Table 1:** Identified aspects for the categorization of situation recognition systems, defined based on [32] and [51]

• Granularity (phases, steps, activities)
• Year
• Sensor data (source) (video (laparoscope), instrument usage (RFID), measurements (OR devices), etc.)
• Method (support vector machines, hidden Markov models, etc.)
• Application area (cataract, laparoscopic cholecystectomy, etc.)
• Usage (online, offline)
• Evaluation (data set) (training, test) (RealOp, SimOp, SimDat (number of cases for training and test))
• Accuracy (possibly precision etc.) (% value)

The category *granularity* was divided into phases, steps, and activities. Because of deviated or inconsistent definitions in and between the papers (e.g., using “task “ or simultaneously “phase” and “step”), our definition based on the definitions of [33, 47] and [38, 45] (see Table 2) was used to classify the papers. The required sensory and overarching methods were extracted for each approach. For the category *sensor data (source)*, the sensor data and data source were identified, but more detailed information (e.g., object to be recognized in the videos, such as instruments via visual features) was left out. For the category *method*, the main techniques (like machine learning and formal methods) were identified while delimiting the highest methods and ignoring details of the recognition. Methods on the feature extraction layer, fine-tuning methods, or similar (e.g., normalization) were largely disregarded, except for unclear boundaries or seemingly important aspects. Abbreviations were used for methods, algorithms, and models (see Table 3). The area of application was determined for the category *application area* according to the data set used for evaluation. For the category *usage*, approaches described as online-capable or respectively intraoperatively applicable (i.e., performance in real-time while only using information obtained up to this point for “live” analysis) were specified as “online.” If defined as real-time-capable, it was assumed that this refers to “online,” unless otherwise stated. If stated as offline, not real-time-capable (i.e., too slow), or data from the entire process was used (i.e., postoperative analysis), it was reported as “offline.” If the online capability was not specified or unclear, the corresponding approaches were defined as “-”. The recognition time (and time for training/model generation) itself was not listed since many papers did not provide detailed information and the numbers are usually not comparable due to different hardware specifications and programming languages used. For the category *evaluation (data set)*, a distinction was made between data sets recorded during real operations (“RealOp”), data sets recorded during simulated operations mimicking real operations (“SimOp”), and artificially generated data sets using manually annotated data, i.e., simulated data provided by the user, also mimicking real operations (“SimDat”). The number of test data and training data (including validation data) was stated in brackets, if identifiable, regardless of whether this data is used several times in changing combinations. If this information is not available, only the total amount of used interventions was stated. The average results of the approaches were expressed in the category *accuracy* as accuracy value in percent. The accuracy was chosen because it is often stated in the machine learning literature as a primary measure of algorithm performance [67]. The definition of accuracy was taken from the respective papers, even though they might have used different definitions on how the accuracy is measured. If no accuracy value was available, the value for, e.g., precision was used and noted as such in brackets. Since many studies compare different approaches (interpretation methods, sensor combinations, and evaluations), the approach with the best result, i.e., the highest accuracy, was chosen. Other results were ignored to avoid the cluttering of the table. Accordingly, only the sensor technology, method, etc. for this result were stated. Approaches for online use and without manual annotation are favored, even if they have lower accuracy.

**Table 2:** Definition of granularities for procedures in this work, defined based on [33, 47] and [38, 45]

Granularity	Definition
Phases	Phases describe the execution of the main objectives of a procedure (e.g., “renorraphy”)
Steps	Steps are taken within the phases to achieve sub-goals (e.g., “cortical suturing”)
Activities	Activities describe the actions performed within steps (e.g., “suture”) and may also contain further information about the specific action, such as anatomical structure and used instrument

**Table 3:** Overview of methods, algorithms, and models used in the papers with abbreviations

Abbreviation	Method/algorithm/model (singular)
AdaBoost	AdaBoost
ATM	Adaptive trace model
BA	Bayesian approach
biLSTM	Bidirectional long short-term memory
BN	Bayesian network
BoW	Bag of words
CCA	Canonical correlation analysis
CNN	Convolutional neural network
CO	Cultural optimization
CRF	Conditional random field
CoRF	Composition of random forests
DT	Decision tree
DTW	Dynamic time warping
GMMAR	Gaussian mixture multivariate autoregressive model
GRU	Gated recurrent unit
HC	Hierarchical clustering
HHMM	Hierarchical hidden Markov model
HMM	Hidden Markov model
HsMM	Hidden semi-Markov model
k-d-tree	k-d-tree
k-Means	k-means
k-Means + +	k-means + +
k-NN	k-nearest neighbor
LSTM	Long short-term memory
MDL	Minimum description length
MIL	Multiple instance learning
MLN	Markov logic network
MM	Markov model
mSVM	Multiclass support vector machine
NB	Naïve Bayes
NN	Neural network
NNS	Nearest neighbor search
OWL	Web ontology language
PCA	Principal component analysis
PKI	Prior knowledge inference
ResNet	Residual network
R(D)F	Random (decision) forest
RNN	Recurrent neural network
SPM	Surgical process model
SQWRL	Semantic query-enhanced web rule language
SWRL	Semantic web rule language
ST-CNN	Spatiotemporal convolutional neural network
SVM	Support vector machine
tCNN	Temporal convolutional neural network

Based on the created table, the presentation and comparison of the strategies were carried out using the defined criteria to show their similarities and differences and to identify trends in research, like favored methods, data sources, and use cases. Therefore, we created partial tables, each focusing on one category of Table 1 (see Online Resource 1, 2, 3, 4, 5). The comparison does not include all properties of the very different approaches (e.g., number of defined phases, modified data set), even if these could be decisive for the results. We neither considered aspects such as the system’s architecture or the detailed functionality. In the discussion, the approaches were discussed within the categories beyond the given aspects of the table. For discussing the best approaches, another partial table was created (see Online Resource 6). In the following section, we focused on the applicability and transferability of the approaches and evaluated their feasibility for achieving situation awareness in the OR during different surgical procedures and used sensor systems. It should be noted that the results of the approaches are based on different evaluation methods and that the comparative results must, therefore, be considered with caution. In contrast to the chapter 3 *Results*, all values for accuracy, performance, etc. were treated as equal for the chapter 4 *Discussion*, while uniformly referring to accuracy.

## Results

A total of 58 papers were identified, covering a wide range of different approaches. Six papers differ in granularity or area and were listed separately (see chapter 3.7 *Approaches with differing aspects*). The remaining 52 papers, summarized in Table 4, are fulfilling all inclusion criteria. Thirty-eight papers have been identified that recognize situations at the granularity of phases, ten papers deal with the level of steps, and four focus on activities in a fine-grained way. The papers are grouped according to their granularity and sorted in ascending order by year. As described in chapter 2 *Methods*, only the best results according to the given definition were shown.

**Table 4:** Overview of identified situation recognition systems

Paper	Year	Sensor data (source)	Method	Application area	Usage	Evaluation (data set)	Accuracy
**Granularity: phases**							
Lalys et al. [34]	2010	Video (microscope)	mSVM, PCA	Pituitary surgery	Offline	RealOp (16)	82.2%
Bouarfa et al. [5]	2011	Video (endoscope, OR camera)	BN, HMM	Laparoscopic cholecystectomy	-	RealOp (9, 1)	90%
Lalys et al. [35]	2011	Video (microscope)	DTW	Cataract	Offline	RealOp (18, 2)	94.8%
Lalys et al. [36]	2011	Video (microscope)	SVM, HMM	Pituitary surgery	Offline	RealOp (16)	93%
Nara et al. [48]	2011	Person trajectories (ultrasound tracker)	MDL, k-means, DT	Neurosurgical tumor resection	-	RealOp (9, 1)	77.18%
Bouget et al. [6]	2012	Video (microscope)	HMM/DTW	Cataract	-	RealOp (20)	94.4%
Weede et al. [63]	2012	Instrument position (tracker), video (endoscope), audio (OR microphone)	NB	Single-port sigma resection	-	SimOp (3, 6)	93.2%
Loukas and Georgiou [42]	2013	Kinematic (electromagnetic tracker)	GMMAR, PCA, k-NN	Laparoscopic cholecystectomy	Offline	SimOp (20, 1)	81.67% (precision)
Charrière et al. [11]	2014	Video (microscope)	NNS	Cataract	Online	RealOp (15, 15)	85.59% (performance)
Katić et al. [27]	2014	Activities (manually annotated)	OWL, SWRL	Laparoscopic cholecystectomy	Online	SimDat (19)	96%
Quellec et al. [52]	2014	Video (microscope)	NNS	Epiretinal membrane surgery	Online	RealOp (23)	87.0%
Quellec et al. [53]	2014	Video (microscope)	CRF	Cataract	Online	RealOp (93, 93)	83.2% (performance)
Stauder et al. [55]	2014	Measurements (OR devices), binary signals object status (OR devices), instrument usage (RFID)	RDF	Laparoscopic cholecystectomy	Online	RealOp (3, 1)	68.78%
DiPietro et al. [15]	2015	Measurements (OR devices), binary signals object status (OR devices), instrument usage (RFID)	SVM	Laparoscopic cholecystectomy	-	RealOp (16, 10)	75.9%
Forestier et al. [16]	2015	Low-level activities (manually annotated), binary signal microscope usage (manually annotated)	DT, HC	Lumbar disk herniation	-	SimDat (22)	87.1% (precision)
Katić et al. [28]	2015	Activities (manually annotated)	SPM, SQWRL	Laparoscopic pancreas resection	Offline	SimDat (11)	90.16%
Quellec et al. [54]	2015	Video (microscope)	MIL, k-NN	Cataract	Online	RealOp (93, 93)	85.6% (performance)
Cadène et al. [8]	2016	Video (endoscope)	ResNet, HMM	Laparoscopic cholecystectomy	Online	RealOp (27, 15)	88.90%
Charrière et al. [10]	2016	Video (microscope)	BN, CRF, k-NN	Cataract	Online	RealOp (25, 5)	82.8% (performance)
Dergachyova et al. [13]	2016	Video (endoscope)	SPM, AdaBoost, HsMM	Laparoscopic cholecystectomy	Online	RealOp (6, 1)	68.10%
Dergachyova et al. [14]	2016	Video (laparoscope)	SPM, AdaBoost, HsMM	Laparoscopic cholecystectomy	Online	RealOp (27, 15)	70.7%
Katić et al. [29]	2016	Activities (manually annotated)	CoRF, CO	Laparoscopic pancreas resection	Online	SimDat (10, 1)	~ 70%
Lea et al. [39]	2016	Video (laparoscope)	ST-CNN, DTW	Laparoscopic cholecystectomy	Offline	RealOp (6, 1)	84.6%
Malpani et al. [43]	2016	System events (daVinci)	tCNN, CRF	Robot-assisted hysterectomy	-	RealOp (23, 1)	76.0%
Twinanda et al. [60]	2016	Video (laparoscope)	CNN, LSTM	Laparoscopic cholecystectomy	-	RealOp (80)	80.7%
Bodenstedt et al. [4]	2017	Video (laparoscope)	CNN, GRU	Laparoscopic cholecystectomy	Online	RealOp (6, 1)	74.5%
Charrière et al. [12]	2017	Video (microscope)	BN, HMM, k-NN	Cataract	Online	RealOp (25, 5)	83.2% (performance)
Stauder et al. [57]	2017	Binary signals instrument usage (OR instruments), binary signals device status (OR devices), measurements (OR devices)	RF, HMM	Laparoscopic cholecystectomy	-	RealOp (17, 1)	82.4%
Twinanda et al. [61]	2017	Video (laparoscope)	CNN, SVM, HHMM	Laparoscopic cholecystectomy	Online	RealOp (40, 40)	81.7%
Volkov et al. [62]	2017	Video (laparoscope)	SVM, HMM	Laparoscopic sleeve gastrectomy	Online	RealOp (9, 1)	92.8%
Jin et al. [23]	2018	Video (laparoscope)	ResNet, LSTM, PKI	Laparoscopic cholecystectomy	Online	RealOp (40, 40)	92.4%
Nakawala et al. [46]	2018	Instrument usage (manually annotated)	SPM, SWRL, OWL	Thoracentesis	-	SimDat (3)	86.25%
Yengera et al. [64]	2018	Video (laparoscope)	CNN, LSTM	Laparoscopic cholecystectomy	-	RealOp (120)	89.6%
Yu et al. [66]	2018	Video (laparoscope)	CNN-biLSTM-CRF, CNN-LSTM	Laparoscopic cholecystectomy	Online	RealOp (80, 40)	83.4%
Hashimoto et al. [21]	2019	Video (laparoscope)	ResNet, LSTM	Laparoscopic sleeve gastrectomy	-	RealOp (88)	82%
Kitaguchi et al. [30]	2019	Video (laparoscope)	CNN	Laparoscopic sigmoidectomy	Online	RealOp (63, 8)	91.9%
Yi and Jiang [65]	2019	Video (laparoscope)	LSTM, ResNet, PKI	Laparoscopic cholecystectomy	Online	RealOp (40, 40)	92.4%
Jin et al. [24]	2020	Video (endoscope)	LSTM, CNN, PKI	Laparoscopic cholecystectomy	Online	RealOp (40, 40)	93.3%
**Granularity: steps**							
Blum et al. [3]	2010	Video (laparoscope)	DTW, CCA	Laparoscopic cholecystectomy	Offline	RealOp (9, 1)	76.81%
Lalys et al. [37]	2012	Video (microscope)	HMM	Cataract	Online	RealOp (18, 2)	91.4%
Padoy et al. [50]	2012	Binary signals instrument usage (manually annotated)	HMM	Laparoscopic cholecystectomy	Online	SimDat (15, 1)	91.6%
Holden et al. [22]	2014	Instrument position (electromagnetic tracker)	PCA, k-means, MM	Lumbar puncture	Online	SimOp (11, 1)	82%
Franke et al. [18]	2018	Device parameter (OR devices SDC), instrument usage (scale), video (endoscope)	ATM, DTW, HsMM	Functional endoscopic sinus surgery	Online	SimOp (23, 1)	94.3%
Zisimopoulos et al. [69]	2018	Video (microscope)	ResNet, LSTM	Cataract	-	RealOp (20, 30)	78.28%
Meeuwsen et al. [44]	2019	Instrument usage (manually annotated)	RF	Laparoscopic hysterectomy	Offline	SimDat (36, 4)	76.8%
Nakawala et al. [47]	2019	Video (endoscope)	CNN, LSTM	Robot-assisted partial nephrectomy	Offline	RealOp (9)	74.29%
Yu et al. [67]	2019	Video (microscope)	CNN	Cataract	-	RealOp (60, 40)	95.6%
Zia et al. [68]	2019	Video (endoscope)	CNN, LSTM	Robotic-assisted radical prostatectomy	-	RealOp (70, 30)	85% (Jaccard)
**Granularity: activities**							
Thiemjarus et al. [58]	2012	Eye gaze (infrared tracker), video (laparoscope), instrument position (infrared tracker)	BN/NN	Laparoscopic cholecystectomy	-	SimOp (15)	93.3%
Lalys et al. [38]	2013	Video (microscope)	mSVM, DTW	Cataract	Offline	RealOp (19, 1)	64.5%
Meißner et al. [45]	2014	Instrument usage (RFID), instrument movement (accelerometer)	HMM, k-means + +	Functional endoscopic sinus surgery	-	SimOp (23, 1)	92%
Twinanda et al. [59]	2015	Multi-view RGBD video (OR camera)	BoW, k-means, SVM	Vertebroplasty	-	RealOp	85.53%

### Sensor data (source)

Various approaches for intraoperative data acquisition exist, through which situations can be identified, either with data sources already available in the OR, such as the endoscopic camera [24], or additional sensor technology, such as RFID [45] or infrared trackers [58]. In the identified papers, different video data [12, 24, 59], information about instruments [50] or persons [48], system events [43], activities [27], or combinations of different sensor data [63] are used. The table shows that the majority of the identified works, 34 papers, uses only video data to detect the current situation in the OR through different features recognized in the videos. Eighteen papers deal with laparoscopic or endoscopic videos. Only real data sets with mostly ~ 40–120 interventions are used. Very different methods are used for interpretation. Thirteen of the approaches are based on CNN or LSTM, which are also combined with each other or with other methods. For phase recognition, 15 cases exist, of which 12 of them use laparoscopic cholecystectomies for application. In the remaining three cases, other laparoscopic interventions are used. Eleven approaches that detect phases can be used online and achieved up to 93.3% accuracy. In the case of step recognition, laparoscopic or endoscopic videos are used in three cases for different applications. There are no online-capable approaches, and a maximum of 85% could be achieved.

Microscopic videos are used in 14 papers. Only real data sets with mostly ~ 20–180 interventions are used. Very different methods are used for detection, which does not indicate preferred methods. Ten papers recognize phases, of which seven concern cataract procedures. The remaining three cases use other scenarios like pituitary or epiretinal membrane surgery. Since 2014, there could always be identified methods for phase recognition that are online-capable, including six papers. These achieved accuracies of up to 87.0%, whereas offline-capable approaches reached up to 94.8%. For step recognition, three approaches exist, which all use cataract surgeries. The only online-capable approach reached 91.4%, whereas the unspecified approaches achieved an accuracy of up to 95.6%. One approach detects activities for cataract interventions. It is not specified as online-capable and only achieved an accuracy of 64.5%.

Two papers use external surgical videos. Only real data sets are used. Both approaches are not specified as online-capable and have different use cases. For the recognition of phases, the video of an external surgical camera is used in combination with the endoscopic video. An accuracy of 90% could be achieved. For activity recognition, the multi-view RGBD video of an external surgical camera is used, where an accuracy of 85.53% could be reached.

Instrument information as the only resource, such as usage or position, is used for situation recognition in six papers. All these approaches use simulated data or data collected during a simulation. Favored methods could not be identified. For phase recognition, only two approaches exist for different scenarios. One of them uses the kinematics captured by electromagnetic trackers, the other approach manually annotated data on instrument usage. Both are not online-capable and achieved up to 86.25% accuracy. Three papers focus on recognizing steps. In two cases, manually annotated instrument usage is used in the area of laparoscopic interventions. The remaining paper uses the instrument position via electromagnetic trackers for the lumbar puncture use case. Two of the approaches are online-capable with an accuracy of up to 91.6%. The approach for activity recognition uses RFID and accelerometers to record instrument usage and instrument movement, respectively. The approach is not specified as online-capable but achieved an accuracy of 92%.

Manually annotated activities are used less frequently for phase detection, just in three cases. The focus here is on laparoscopic interventions, whereby different methods are used. Two of the approaches can also be used online and achieved an accuracy of up to 96%. Only one paper deals with personal data as the data source in form of person trajectories collected by ultrasound trackers. Another paper uses device data, system events of the daVinci surgical system.

Combinations of different data sources, such as device and instrument data, are also used more often, in seven papers. Both real data sets and simulated data are used. For detection, different methods are used. In the case of phase recognition, a combination of different data types is used in five cases, of which three use the application area laparoscopic cholecystectomy. Two of them use measurements and binary signals of the object status of devices as well as the usage of instruments via RFID. The other paper uses binary signals of instrument usage as well as binary signals of the device status and measurements of devices. The remaining two papers use the instrument position of trackers, the endoscopic video, and the audio from a surgical microphone or manually annotated low-level activities and the binary signal of microscope usage, respectively. Just one of the five approaches is online-capable, achieving an accuracy of 68.78%, while the remaining unspecified approaches reached up to 93.2%. The only paper to recognize steps uses device parameters via SDC, the instrument usage via a scale, and the endoscopic video as input. With this online-capable approach, an accuracy of 94.3% could be achieved. For recognizing activities, just one paper exists that uses a combination of the eye gaze and the instrument position of infrared trackers as well as the laparoscopic video. The approach is not specified as online-capable but reached an accuracy of 93.3%.

### Application area

Twenty-seven approaches deal with laparoscopic cholecystectomies (21 cases) or other laparoscopic procedures (six cases). For these, video data is mostly used, in 17 cases, but also other sensor data, such as instrument data, solely or in combination with device data, is applied in seven cases. Real data sets of interventions are predominantly used, despite of seven cases, often comprising ~ 20–120 interventions. Very different methods are used for recognition. Eighteen of the approaches are based on CNN, LSTM, or HMM, which are also used in combination with each other or with other methods. Fifteen of the approaches can be used online and achieved accuracies of up to 96% by detecting phases, whereas an accuracy of 91.6% could be reached in the case of recognizing steps. The only approach for activity recognition is not defined as online-capable and achieved an accuracy of 93.3%.

Many approaches, 11 cases, focus on cataract procedures that use microscopic videos exclusively. Only data sets of real interventions with ~ 20–180 interventions are used. A preferred method used by these approaches cannot be determined. The identified methods for phase recognition since 2014 are all online-capable. These methods achieved an accuracy of up to 85.6%, whereas 94.8% could be reached offline. Similar for step recognition, online an accuracy of 91.4% could be achieved but 95.6% by an approach not specified as online. Activity recognition reached only 64.5% with an offline-capable approach.

Other interventions are also used for evaluation, twice pituitary surgeries, twice functional endoscopic sinus surgeries, and ten other surgeries. The input data varies widely, from video data to instrument information to combinations. For pituitary surgeries, e.g., just the microscopic video is used. Real data sets are used in eight cases but also simulated data or surgeries. Besides, different methods are used that do not indicate favorites. Only three of the approaches can be used online. For phase detection, online accuracies of up to 87.0% can be achieved but for an unspecified approach 93.2%. For the recognition of steps, online 94.3% can be reached, for the recognition of activities up to 92% for non-online-capable approaches.

### Evaluation (data set)

Thirty-nine out of 52 papers use data sets of real interventions. The remaining works simulate interventions (six cases) or use simulated, manually annotated data (seven cases). The data sets used in the papers contain different naming and amount of situations (i.e., papers recognize their selected phases) as well as different use cases. The amount of data varies a lot. Sixteen of the approaches with real data sets use a total of more than 40 interventions, 12 less than 20 interventions. Since 2018, the approaches for recognizing phases always use more than 70 interventions. Overall, different methods are used, whereby approaches based on CNN, LSTM, HMM, or SVM are listed in 30 cases, which are also used in combination with each other or with other methods. Mostly, all of these papers, 34 approaches, are based on video data; 31 have the use case laparoscopic surgery, especially laparoscopic cholecystectomy, or cataract surgery. About half of the approaches are online-capable. With these, accuracies of up to 93.3% and 91.4% could be achieved in the detection of phases and steps, respectively. For unspecified approaches, an accuracy of 94.8% for phases and 95.6% for steps could be achieved.

The approaches that use simulations of real interventions for evaluation all contain less than 25 interventions. Favored methods could not be identified. The papers use different data sources, but always instrument data is included as input. The use cases are different. Online, only those approaches that recognize steps can be used which achieved an accuracy of up to 94.3%. Unspecified approaches in case of online capability reached an accuracy of up to 93.2% or 93.3% for phases or activities, respectively. Approaches that use manually annotated data as data source evaluate with less than 20 interventions, despite of two exceptions. Different approaches without recognizable focus are used. The approaches focus on manually annotated activities or instrumental usage to interpret the current situation. Laparoscopic interventions were chosen in five cases. Online, the approaches achieved up to 96% accuracy in phase recognition and 91.6% in step recognition.

### Usage

Many of the approaches are not designed for intraoperative use but for postoperative purposes. Twenty-four approaches can be applied online, whereas ten of the approaches can only be used for offline detection. Eighteen papers do not specify this aspect at all. To detect the current situation in the OR online, real data sets are used in 19 cases, mostly with ~ 20–120 interventions. Although various combinations of methods are used, approaches based on HMM or CNN are listed most frequently (13 cases). For recognition, the majority of papers, 18 approaches, uses video data, which is why the focused use cases are laparoscopic and cataract interventions, despite of three exceptions. Phase recognition achieved an accuracy of over 90% in six cases, especially since 2018, with the best result reaching 96%. For the recognition of steps, almost all approaches, despite of one, achieved accuracies above 90%, with the best result being 94.3%.

For offline recognition, seven papers chose real data sets, three simulated data. The methods used are very different. Video data is also preferred (seven cases), and laparoscopic procedures are used in half of the approaches for evaluation. The approaches achieved an accuracy of up to 94.8% for detecting phases, whereas steps can be detected with a maximum of 76.81% and activities with 64.5%. The approaches that are not defined as online or offline predominantly use data sets from real interventions, 13 cases, often comprising ~ 20–120 interventions. Specific methods are not favored. The approaches include more variance in terms of data sources. Video data, as well as combinations, are used for 15 approaches. The approaches are evaluated in different interventions, seven times laparoscopic, but also in many other areas. For phase recognition, accuracies of up to 94.4% can be achieved, for steps 95.6% and activities 93.3%.

### Accuracy

The accuracy of the approaches ranges from 64.5 to 96%. Newer approaches are not necessarily better than older ones. The majority of the papers, 39 approaches, achieved a higher accuracy or comparable unit of over 80%. The best 18 approaches with 90% or more use different methods, whereby approaches based on HMM are used in eight of the cases, also in combination with other methods. For the detection of phases, real data sets are used, despite of three cases, which contain different numbers of interventions. In eight cases, the use case laparoscopic intervention is focused. Nine of the papers recognize only via video data. Six of the approaches are online-capable for which accuracies of up to 96% were achieved. Since 2017, online-capable approaches could always be identified. For the recognition of steps and activities, no favored data source or use case could be identified. More simulated than real data is used (four vs. two cases). Steps were predominantly identified online, with one exception, with up to 94.3%, whereas the only unspecified approach even reached 95.6%. Activities were detected with up to 93.3%, whereas it is not defined whether the approaches are online-capable. The approach with the highest accuracy considering all granularities is online-capable and based on manually annotated activities of laparoscopic cholecystectomies, where phases are recognized via OWL and SWRL.

Twenty-one approaches achieve accuracies of 80 to 90%, 17 of them are evaluated on real data sets. Approaches based on CNN or LSTM could be identified in seven cases, which are also frequently used in combination with each other or with other methods. As a data source, video data is used conspicuously often, in 16 cases. The use cases laparoscopic procedures and also cataract procedures are used in 14 cases. Ten of the approaches are online-capable.

### Method

There are two main approaches for situation interpretation: machine learning techniques and formal methods (e.g., ontologies) for machine-readable modeling of medical knowledge [26]. Machine learning includes algorithms that analyze the data to build a statistical model that is then used to identify unknown aspects. The learning techniques are trained in advance using data sets. The trained models can then be used with sensor data as input (e.g., instrument position) to provide an output that reflects the situation in different granularities. Formal methods, including ontologies or surgical process models (SPM), can be used to represent knowledge about the process or other relevant aspects and use logical links and rules for the interpretation of the situation.

In literature, for used methods, no clear trend could be identified. But it is noticeable that some approaches rely on only one method, while other approaches use a combination of different methods. Particularly in the detection within videos, additional methods are often used to detect features in the image data which have been left out for the overview as far as possible. Overall, machine learning techniques and formal models both are used for approaches, partly both in one approach. It could be identified in the individual categories that methods based on CNN, LSTM, HMM, or SVM (solely or in combination with various methods) are used more frequently, in 33 cases.

### Approaches with differing aspects

Six papers were listed separately due to deviating granularity and application (see Table 5). Three of them recognize only very few intraoperative phases, but more pre- and postoperative phases; one uses states (e.g., “risky situations”). The remaining two papers, and two of the papers mentioned before, deal with trauma resuscitations that do not represent an intervention.

**Table 5:** Overview of identified, but differing, approaches concerning situation recognition systems

Paper	Granularity	Year	Sensor data (source)	Method	Application area	Usage	Evaluation (data set)	Accuracy
Bardram et al. [2]	Phases (few intraoperative)	2011	Person location (RFID), instrument/object location (RFID), instrument/object usage (RFID)	DT	Laparoscopic appendectomy	Online	SimOp (3, 1)	77.29%
Katić et al. [26]	Phases (states)	2013	Instrument position (optical tracker)	OWL, BA	Laparoscopic cholecystectomy	Online	SimOp	97%
Li et al. [41]	Phases (few intraoperative)	2016	Depth video (OR camera), audio (OR microphone)	CNN	Trauma resuscitation	Online	RealOp (20, 5)	80%
Gu et al. [20]	Phases (few intraoperative)	2017	Audio (OR microphone)	LSTM	Trauma resuscitation	Offline	RealOp (24, 3)	41.13%
Chakraborty et al. [9]	Steps	2013	Video (OR camera)	MLN	Trauma resuscitation	-	SimOp (10)	91.14% (precision)
Li et al. [40]	Steps	2016	Object usage (RFID)	CNN	Trauma resuscitation	-	RealOp (16)	80.40%

Good results could also be achieved by these approaches. For evaluation, real and simulated data are used. The methods used are different. Four of the papers deal with the recognition of phases. For recognition, different data sources are used. In one case, RFID technologies for person location, instrument/object location, and usage are used. Another paper uses optical trackers to obtain the instrument position. The remaining two papers use the audio of a surgical microphone, solely or in combination with the depth video of an external surgical camera, respectively. Of three online-capable approaches, the best accuracy was 97%. On the granularity of steps, an external surgical video or the object usage via RFID is used as input for recognition. Up to 91.14% was achieved with not as online-capable defined approaches.

## Discussion

The review shows that there are many approaches with good recognition rates. In the following, these are discussed beyond the table and afterward evaluated for their applicability and transferability to other scenarios and data sources.

### Sensor data (source)

The table shows that up to 96% accuracy can be achieved for the seven cases which are using manually annotated data from activities, microscope usage, or instrument usage. Overall, the results are usually over 85%. Certain work beyond the table can show that the use of manually annotated data, sometimes with a different method, can provide an improvement in accuracy. Dergachyova et al. [13], for example, can increase accuracy from 68.10 (video only) to 88.93% by combining visual and instrument information, using manually annotated data on instrument usage in addition to video data. Similarly, Lea et al. [39] show that if manually annotated data on tool usage is used in addition to video, the accuracy can be increased from 84.6 to 92.8%. The multi-level approach of Charrière et al. [10] shows that compared to the use of video data with 82.8% for phase detection, better results of 98.6% can be achieved with manually annotated instrument data. Similarly, Charrière et al. [12] show that 83.2% can be reached for phase recognition using video, whereas 98.6% could be achieved with manually annotated instrument data. Yu et al. [67] reached an accuracy of 95.6% with videos, while manually annotated instrument usage data can increase the accuracy to 95.9%. Gu et al. [20] (approach with differing aspects) describe a method that achieved 41.13% with audio recordings and 79.12% with manual transcriptions. All these results are due to the fact that errors can already occur during the recognition of instruments etc., which in turn affects the recognition of the current situation. With manually annotated data, these errors do not occur, so approaches can work with perfect data. If the data were collected via intraoperative sensors, worse results can be expected.

Video data is chosen for more than half of the approaches. Instrument data is the second most used data source, partially in combination with device or other data. The accuracies for these are varying. A tendency for single data sources or a combination of data to yield better results could not be observed. Very good results can be reached with only one data source or with a combination of data sources, without focusing on a specific source. In any case, it seems reasonable to include all available inputs for situation recognition. The most promising approach would probably be to integrate as many sensors as possible to take into account inaccuracies of a single sensor and to make the analysis more robust [31].

### Application area

The approaches seem to be designed for a specific intervention and are specially trained for it. Laparoscopic and cataract procedures are focused in research. For these, types of data sources are focused. For example, cataract procedures only used the microscopic video, and laparoscopic interventions focus on the laparoscopic/endoscopic video. Overall, the application area is conspicuously set on standardized interventions or seems to provide the best results for them.

### Evaluation (data set)

Although the individual approaches often show a high degree of accuracy, the evaluations were not carried out during an intervention; either data sets from real interventions or simulated data/interventions were used. The live application was not tested extensively. The data that was recorded during interventions was also used for testing the approaches. No live evaluation in the OR was carried out, which questions the functionality. Some of the studies also describe that the approaches provide good results but are not yet good enough for clinical use. For example, the approach of Franke et al. [18] is suitable for laboratory conditions but must be extended for clinical use. Above all, some approaches cannot be integrated into the OR at all, as they are based on manually annotated data, i.e., they do not use real sensor data for interpretation. To use them, the input data first have to be determined automatically, for example, by intraoperative sensors (e.g., [27, 28]). Also, the number of data in the data sets used should be increased so that more variance can be represented within the data set and the approaches can be extensively trained and tested.

### Usage

Offline approaches can often reach better accuracies, because, for example, the entire video can be used for analysis instead of only using the video available up to the time of the operation (which is the case for online usage). This tendency is not visible in the tabular presentation. Very good results can also be achieved online. However, in Twinanda et al. [61], it is shown that an accuracy of 81.7% can be reached online, whereas offline even 92.0% can be achieved. Similarly, Lalys et al. [37] show that an HMM-based approach reached an accuracy of 91.4% online, while a DTW-based approach achieved 94.4% offline. It can be concluded that it does make a difference whether the approach is used online or offline. But only online-capable systems can be used for situation recognition during procedures. Approaches that are not defined as online-capable can probably not be used live. Offline approaches could be used for postoperative analysis and still be extended for online capability in future work.

### Accuracy

According to the table, the approaches cover a wide range of accuracies. Specific conditions for achieving the best results could not be identified, because of the very different approaches regarding the combination of sensor data, method, area, etc.

### Method

The table shows that models defined as SPM are used by four of the identified papers, which achieved an accuracy of up to 90.15% but do not stand out overall. Two of the approaches have a lower accuracy of 70%. Ontologies are also used in six cases, although not stated in the table, some of which reached an accuracy of more than 90% but do not particularly stick out as well. The use of SPMs in combination with ontologies was also observed in two of the six ontology cases. Nakawala et al. [46] use an ontology to represent knowledge about thoracentesis interventions. For the interpretation, rules based on SWRL and OWL are created to realize an SPM. The approach achieved an accuracy of 86.25%. Katić et al. [27] use a rule-based situation interpretation using OWL and SWRL. The workflow of the interventions is formalized by an ontology. For laparoscopic cholecystectomies, an accuracy of 96% was achieved. Katić et al. [28] present a rule-based situation interpretation using SQWRL. The workflow of the interventions is formalized by an ontology to represent an SPM. In the use case laparoscopic pancreas resection, an accuracy of 90.16% was achieved. Katić et al. [29] combine formal knowledge via an ontology with experience-based knowledge. The approach is based on CoRF and CO and achieved an accuracy of ~ 70% for laparoscopic pancreas resections. Lalys et al. [38] use an ontology, mSVM, and DTW to detect activities based on previously detected phases to automatically generate SPMs of cataract interventions. An accuracy of 64.5% was achieved. Katić et al. [26] (approach with differing aspects) present a combination of machine learning and formal methods, which uses OWL and BA in addition to an ontology-based situation interpretation. An accuracy of 97% could be achieved in the scenario laparoscopic cholecystectomy. Although the papers that use SPMs and/or ontologies are not among the best overall, as there are both good and worse results, it seems to be useful to use SPMs and ontologies to represent knowledge about aspects of the process and to use this knowledge for situation awareness. A combination of machine learning techniques and formal methods seems reasonable.

### Best approaches

To identify clearer trends in the presented papers, the best approaches were defined. These include an accuracy above 90%, the possibility of online usage, and the use of non-manually annotated input data. Seven approaches could be identified with this criteria. It is noticeable that only endoscopic or laparoscopic videos are used for phase recognition (five approaches in total). With laparoscopic interventions as the application area, the best results were achieved from 91.9 to 93.3%. Real data sets with ~ 70–80 procedures were used, except one with only ten procedures. Mostly combinations of methods were used, two of the studies use an approach based on ResNet, LSTM, and PKI. The best approach uses LSTM, CNN, and PKI. For step recognition, no laparoscopic procedures are used but cataract and functional endoscopic sinus surgery (two approaches in total). For the cataract use case, the microscopic video is used, for the other case, in addition to the endoscopic video, device parameters via SDC and information on instrument usage via scale. The accuracies were 91.4% and 94.3%, respectively. For the first approach, a real data set with 20 interventions was used, for the second 24 simulated interventions. No comparisons could be made between the methods. The better approach uses ATM, DTW, and HsMM. The detection of activities could not keep up with the results of the other two granularities.

Video data is always used for both phase and step recognition to achieve the best accuracies. This seems to be a suitable source of data but can only be used for certain interventions that capture such videos. Only one approach uses other data sources in addition, which ultimately resulted in the highest accuracy. This approach is the only one that uses simulated interventions, which is therefore not necessarily meaningful. Overall, the approaches are very current (since 2017), with one exception of 2012. The best approach for phase and step detection is always the most recent one. Favored methods could not be identified unambiguously, but combinations of different methods seem to make sense, as the lowest of the best accuracies are also achieved by those approaches with only one method for interpretation.

### Differing approaches

The best result by approaches with deviating aspects with an accuracy of 97% is reached when detecting states. For this purpose, the instrument position is recorded via optical trackers and interpreted via OWL and BA. The approach is online-capable, but the results were obtained by simulated laparoscopic cholecystectomies. The second best approach achieved 91.14% in the detection of steps in trauma resuscitation. Simulated procedures are also used, with surgical videos as input for recognition and MLN for interpretation. The approach is not defined as online-capable. The approaches show that good solutions also exist in non-focused areas, which can provide good results in deviating use cases. It may be useful to extend these to desired conditions (intraoperative use).

### Concluding remarks

Overall, the table results and discussion only reflect trends, as not all characteristics of the very different approaches can be included. Depending on the case, the data sets may contain different defined phases etc., which may also vary in number. There is no clear definition of the phases, but the research groups define them themselves or they were determined based on existing data sets (number and allocation of activities to a phase varies). In addition, due to the focus on approaches with online usage and without manually annotated data, an increasing number of such approaches are listed. Due to the different evaluation methods, the results are not directly comparable. The comparison results must, therefore, be considered with caution. Nevertheless, it is recognizable that the approaches cover a broad spectrum of methods and sensors. There is a clear tendency towards video data and the corresponding use cases. However, it does not show that certain methods or sensor data only provide good results but rather that very different approaches are among the best, which do not all use the favored data sources or application cases.

### Applicability and transferability

Within the different studies, the approach with the best results is not always favored. Charrière et al. [12] are an example of this. In this paper, a different approach than the one with the best accuracy is recommended, because it can be transferred to other video-monitored interventions and levels. From this, it can be concluded that accuracy is not always the means to measure the best solution. We assume that applicability and transferability to other processes, granularities, and sensor data are also important. The importance of the transferability of strategies within the OR is addressed, for instance, in [19]. This paper discusses that many instrument recognition systems focus on specific interventions and are not generally applicable. Instead, a system that can be used for a large number of operations is defined as necessary [19]. Deducing from this, it is necessary for the broad application of a situation recognition system that it can be easily adapted to different conditions, so that it can be used for a majority of interventions, independent of available data and planned support.

#### Papers that hypothesize transferability

Applicability and transferability of the approaches are not addressed in all papers. Regarding the flexibility of the sensor technology used, the comparison of the identified systems showed that the approaches are designed for specific sensors. Concerning the usability of sensor sources, the choice often falls on endoscopic, laparoscopic, or microscopic videos, as these are already available in the OR. Using video data avoids the need to install additional equipment in the OR and provides a source of information that must not be controlled by humans, thereby automating the support of surgeons without the need to change the surgical routine [37]. In contrast, other data sources, such as RFID tags or different trackers, require that they were attached to instruments, devices, and people, which can be considerably more complex (e.g., if each instrument has to be equipped with an RFID tag). For using extra cameras or other elements (e.g., scales), they must be installed or integrated in the OR as well. The cost-effectiveness and feasibility of such strategies, which, for example, require modification of instruments, are questionable [19]. Approaches that use video data already available in the OR allow for simpler applicability and transferability, because no change of equipment or processes is necessary. These approaches can theoretically be transferred but only to other video-based interventions, without the need to integrate additional sensors, provided that the method has been trained for them and knowledge about processes and situations exist. Very similar, solutions that use device information such as system events or measurements can be transferred as well if the data is available in the respective OR. However, there is a lack of available and suitable medical device data due to the lack of open communication standards [25]. Not in every OR certain technologies are integrated that can be used to collect data. For interventions for which no video data or sufficient device data are available, additional sensors must be attached for transmission. Moreover, many studies show that additional integrated sensors can achieve good results (e.g., RFID and accelerometers [45]). For as little effort as possible, sensors that are easy to integrate could be used (e.g., [15]).

In addition to the focus on video data, it is also very noticeable that the identified approaches deal with highly standardized interventions. The approaches for these are often designed for a specific intervention and are specially trained for defined phases etc. For individual use cases, there also exist extensive data sets for this purpose, with which the methods can be trained (e.g., using video data). The transferability of the approaches to other interventions is difficult to assess. A 1-to-1 transfer seems very unlikely since interventions can vary greatly (e.g., steps to be performed, instruments used), and therefore, it cannot be guaranteed that a trained approach can be used for other processes in the same way with similar results. In principle, this is possible with adjustments by training the methods for the new interventions and, if necessary, additionally mapping the required knowledge about the new processes (incl. data). Thus, the approach can be trained for other defined phases etc. For more complex, variable interventions, the recognition does not seem to be successful so far due to the non-standardized and flexible processes. These are often unpredictable and make it difficult to define knowledge about the process that can be used to identify the situation. The processes cannot be modeled in a structured way, several data combinations may be possible, and therefore, this knowledge cannot be incorporated into the detection (e.g., dependencies between steps). Thus, many approaches are specialized in highly standardized interventions, which can be better analyzed based on the clear procedures and the often unambiguous assignment of data to situations. The definition of the workflow is only feasible for interventions if their procedure is standardized [32]. A transfer to more variable processes is therefore hardly possible.

The usability of the approaches for other processes is only addressed to a limited extent in the identified papers. For approaches that work with laparoscopic, endoscopic, or microscopic videos, it is more often described that these are, for example, generalizable or scalable and can therefore also be used for other types of interventions (e.g., [13]), more complex procedures (e.g., [4]), or other data sets (e.g., [61]). Rarely, the transferability to other granularities or sensor sources is mentioned. Nevertheless, some papers state that the approach can be adapted to other levels (e.g., [10]), be used in a more fine-grained way (e.g., [62]), or be extended to other sensor data (e.g., [47]). Also when using other data sources, approaches are described as generalizable or scalable and are therefore applicable to other procedures (e.g., [50]) or different settings (e.g., [9]). The detection of different granularities or expandability of sensors is also rarely shown. Few papers describe that the approach is transferable to other levels of granularity (e.g., [16]) or can be extended by sensors (e.g., [41]). Other studies indicate that their approach is not well transferable to other interventions or data sets, for example, in the case of more variable data (e.g., [39]). Additionally, few papers define their approaches as generalizable or extendable but not necessarily applicable to other areas in their actual form, because cues are application-dependent (e.g., [52]) or the approach needs to be trained for each department (e.g., [35]).

#### Papers that demonstrated transferability

Rather rarely, the transferability of the approaches to other data sets or use cases is demonstrated in tests or experiments. Some papers apply their approach to different data sets, which may also include other use cases or different methods. For example, Jin et al. [23] compare the results for two data sets of laparoscopic cholecystectomies, with slightly different accuracies of 90.7% and 92.4%. Twinanda et al. [60] use the same two data sets and achieved 79.5% with one data set and 80.7% with the other. Lea et al. [39] use two other data sets with laparoscopic cholecystectomy procedures, but the results are very different, 63.7% with one and 84.6% with the other. Quellec et al. [52] show results of two data sets, including different use cases, epiretinal membrane surgeries, and cataract surgeries, which reached 87.0% and 72.9%, respectively. Bodenstedt et al. [4] also use two data sets with different cases, laparoscopic cholecystectomies, and colorectal laparoscopies, which achieved 74.5% and 67.2%, respectively. Katić et al. [27] even use three different data sets for the scenarios laparoscopic cholecystectomy, pancreatic resection, and adrenalectomy which achieved 96%, 90%, and 83%, respectively.

These examples show that when approaches are applied to different data sets, the accuracies can vary greatly, especially when data sets from different interventions are used. If data sets of the same procedures are used, very similar results could be observed in some cases, although a larger difference can occur here as well. The examples seem to show that one approach cannot be used equally well for other data sets. This is probably due to the differences in the data (e.g., detecting other phases) and to the different process flows (e.g., more complex interventions or ambiguous assignment of data to situations). The data set and thus the use case seem to have a strong influence on the recognition accuracy. The amount of training data will also influence the results. For future approaches, more complex evaluations should be carried out under real conditions [51]. Furthermore, more experiments with more complex and variable interventions should be done.

The transferability of the approaches to other granularities or the recognition beyond one granularity is also rarely shown practically. Nakawala et al. [47] show that, beyond the already recognized step, an ontology-based SPM and rules can identify complementary context (activities, phases, and instruments) based on the step. However, further context could be identified with less accuracy, since the recognition is based on the recognized step. Lalys et al. [38] identify activities based on previously recognized phases, whereas Franke et al. [18] identify steps based on previously recognized activities. Charrière et al. [10] use phases and steps and their influence on each other for multi-level recognition, whereby phases could be recognized with higher accuracy. Similarly, Charrière et al. [12] use knowledge of the relationship between phases and steps to identify situations. Again, the results for phase recognition were better. The examples show that the granularities are interdependent. Beyond one granularity, further knowledge about other contexts can be identified. Furthermore, the granularities seem to be able to influence each other, through which multi-level recognition can benefit. The transferability to other granularities thus seems to be possible in principle via knowledge of the interventions.

Even less frequently, the transferability of the approaches to other sensor data is demonstrated in the papers. A flexible connection of available sensors would result in independence so that transferability to new ORs with other sensor technology is possible. In different works, the results using different sensor data are compared. Malpani et al. [43] show, for example, that a subset of features, in contrast to the system events of a daVinci surgical system, can be used for many interventions (laparoscopic, endoscopic, and open) and can also deliver comparatively good results, only slightly worse with 72.5% instead of 76.0%. DiPietro et al. [15] show that with fewer, rapidly deployable sensors, very good results can be achieved (73.9%), whereas more sensors could reach 75.9%. The examples make it clear that for transferability, it makes sense to choose data sources that can be used for a variety of interventions or that are easy to integrate. So approaches can be used not only for one intervention, for example, by using a specific device. With current techniques, this seems to be possible only to a limited extent.

#### Concluding remarks

Most papers do not address the transferability of their approaches nor demonstrated it based on different data sets. In these cases, it was assumed that they are not transferable. Some papers state that the approaches are generalizable, but they do not describe in detail or demonstrate only slightly how the individual approaches can be transferred exactly to other interventions, phase definitions, granularities, or sensor data. The approaches always seem to be limited in terms of training data set (type of intervention), process modeling, sensor technology, and other aspects. The methods are trained based on a data set and must, therefore, be trained for the new application. In addition to training based on a new data set, new process models or similar may be necessary. Furthermore, different situations (e.g., additional phases, data sources) must be considered. For easy transferability, all this must be given. Therefore, the approaches, although described as generalizable, do not seem to be used easily without additional effort, because these aspects are not addressed in this way. The papers that show applicability and transferability usually use only two different data sets for evaluation. For this purpose, more different data for sufficient tests or respectively the application directly in the OR would be advantageous. The differences in detection accuracy also make it clear that transferability is possible, but the results can vary greatly. For very similar interventions, simple transferability might be possible without having to adapt the entire approach, but for different interventions, the approaches have to be adapted and, if necessary, extended (training, knowledge, data, etc.).

## Conclusions

The identified approaches differ in many aspects, such as method, area, or accuracy. The focus is clearly on the use of video data for standardized use cases such as laparoscopic and cataract surgery, although not all of them necessarily achieve good results. Videos are probably preferred as they are already available in the OR. In addition to video, instrument data is also quite often used solely or in combination with other sources. The works are often based on data sets from real interventions but sometimes use only a small amount of data. More data should be used for training and testing. We assume that broader availability of annotated intervention recordings with a broad range of sensor input would be a significant contribution to this research field. Furthermore, the approaches should also be tested live. Often the approaches can be used online, but this is not always stated. During surgery, only approaches that are online-capable can be used, which limits the number of possible systems for intraoperative use. Nevertheless, many online approaches already show very good recognition results. The accuracy of the strategies varies as much as used methods. Many of the studies show very good results with accuracies above 80% or even 90%. However, no clear trend could be identified for methods, although combinations of different methods and the usage of machine learning combined with formal methods seem to be useful.

The discussion of the papers concerning applicability and transferability showed that the approaches can be used in principle to achieve situation awareness about intraoperative processes. Transferability is less addressed in the papers and is hardly ever demonstrated by experiments. Nevertheless, especially the approaches based on video data seem to be transferable to other video-based interventions due to the availability of the data source. Therefore, flexibility in adapting to the changed processes must be guaranteed. The transferability to other processes, granularities, and data sources is only outlined in a few papers and seems to be possible only to a limited extent. Although some studies mention that their approaches can be generalized to other types of interventions etc., they do not show this enough in experiments. Through a few examples, it could be shown that different data sets can strongly vary in recognition. A few studies demonstrate that their approaches can be used beyond a granularity or a specific sensor source combination. The recognition of more context and also the adaptability with regard to sensor data is an important step for the broad usability of such a system. Therefore, future work should focus more on aspects of applicability and transferability to make recognition systems more adaptive. The goal for future developments should be much more on the broad applicability of solutions to reduce highly specific systems for a minimum of interventions. For this purpose, it is recommended to make the system easily adaptable to different circumstances by, e.g., supporting different sensor data sources and application areas. We assume that a unified solution that can be adapted to different processes and granularities of the intraoperative area and that robustly detects the current situation in the OR without requiring specific sensor technologies would allow for greater flexibility, applicability, and thus, transferability to different applications.

## Supplementary Information

Below is the link to the electronic supplementary material.

Online Resource 1: Identified situation recognition systems grouped by Sensor data (Source) (XLSX 46 KB)

Online Resource 2: Identified situation recognition systems grouped by Application area (XLSX 45 KB)

Online Resource 3: Identified situation recognition systems grouped by Evaluation (XLSX 28 KB)

Online Resource 4: Identified situation recognition systems grouped by Usage (XLSX 28 KB)

Online Resource 5: Identified situation recognition systems grouped by Accuracy (XLSX 32 KB)

Online Resource 6: Identified situation recognition systems showing best approaches by accuracy, usage and sensor data (XLSX 19 KB)
